# Supplementary material for: Food–Drug Interactions: Effect of Propolis on the Pharmacokinetics of Enrofloxacin and Its Active Metabolite Ciprofloxacin in Rabbits
Source: Pharmaceuticals (Basel). 2025 Jun 27;18(7):967. doi: 10.3390/ph18070967 (PMC12299926; doi:10.3390/ph18070967)
Supplement: Supplementary file 1 [file pharmaceuticals-18-00967-s001.zip › pharmaceuticals-3705453-supplementary.pdf]

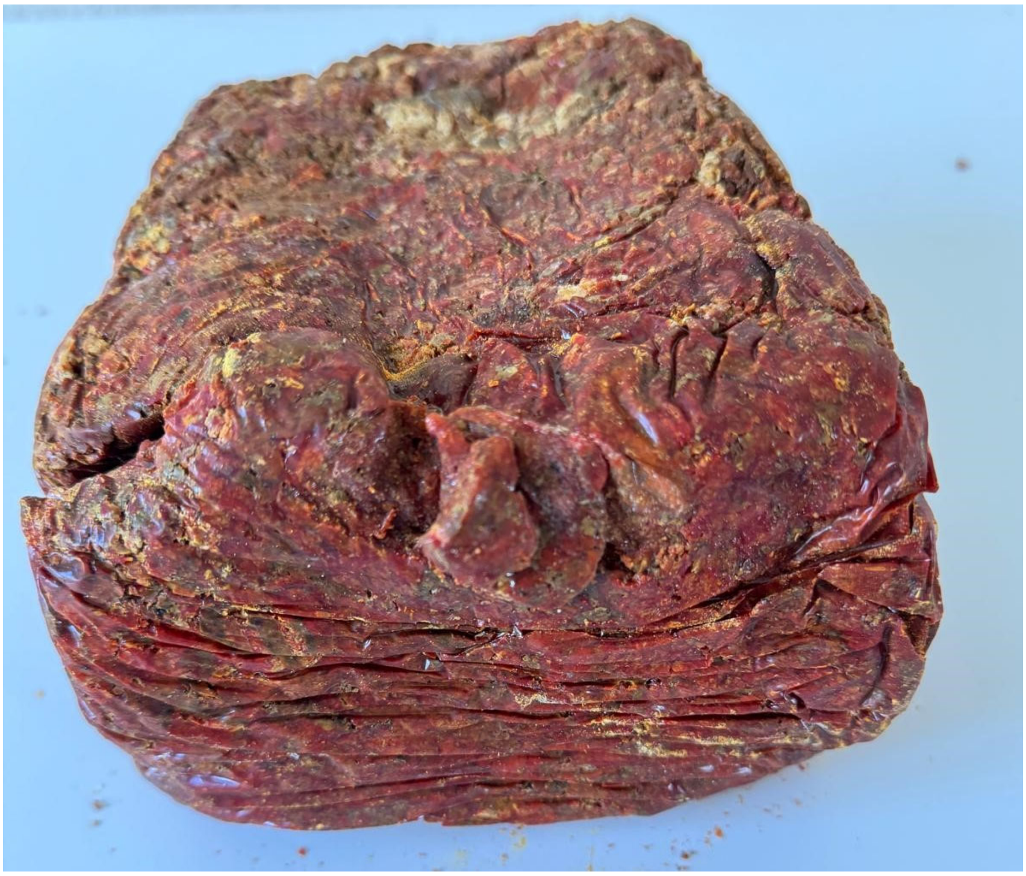

Figure S1. The reddish propolis sample which was collected.

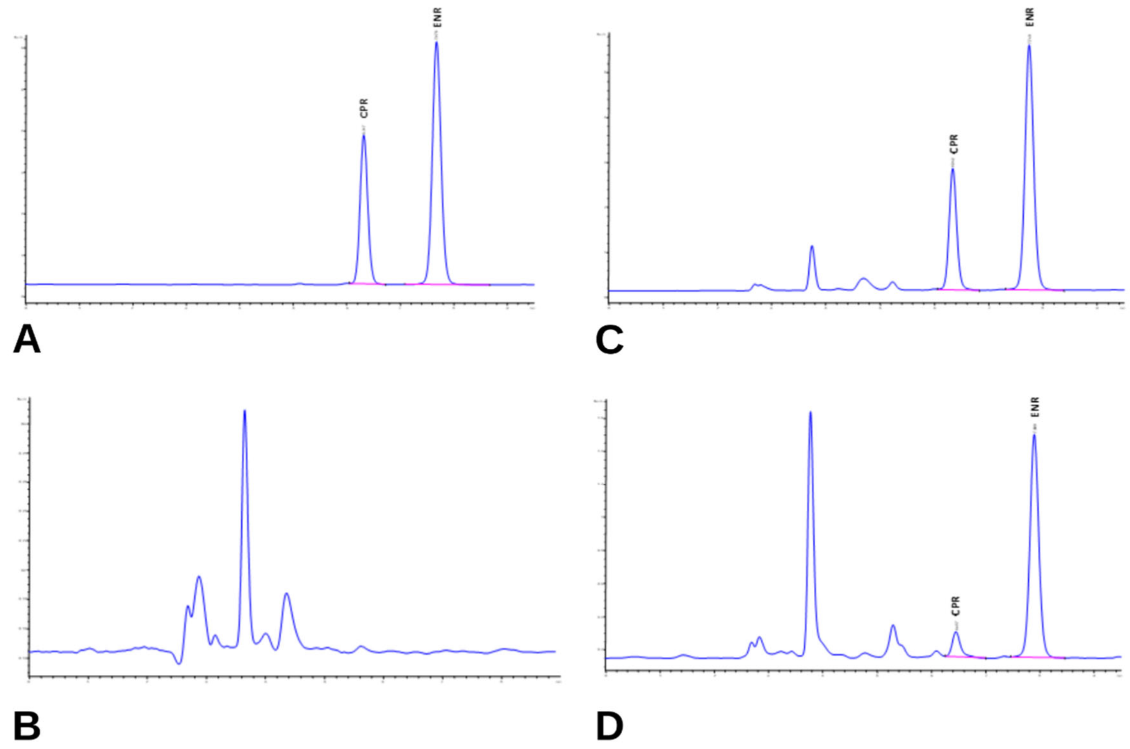

Figure S2. The chromatograms of enrofloxacin and ciprofloxacin by HPLC-FLD.

Analytic standard mixture of ENR and its metabolite CPR (A, 0.5 μg/ml), a blank plasma sample (B), a fortified plasma sample (C: 0.5 μg/ml) and a 12-h experimental sample following i.m. administration of ENR in rabbits (D).

Table S1. Mean plasma concentration (μg/ml) of ENR and CPR HPLC-UV analyses after IM. ENR and IM ENR+ propolis (PP) administration.

T (h)	ENR		ENR+PP		CPR		CPR+PP	
	Mean	SD	Mean	SD	Mean	SD	Mean	SD
**0.0**	0	0	0	0	0	0	0	0
**0.1**	1.498	0.720	1.803	0.656	0.003	0.009	0.006	0.010
**0.3**	2.699	0.531	2.583	0.412	0.042	0.020	0.043	0.020
**0.5**	2.942	0.379	2.670	0.540	0.107	0.025	0.070	0.027
**1**	2.479	0.464	2.636	0.426	0.169	0.021	0.123	0.056
**2**	1.819	0.280	2.221	0.302	0.206	0.026	0.176	0.065
**4**	0.946	0.181	1.490	0.419	0.169	0.025	0.171	0.044
**8**	0.374	0.132	0.767	0.360	0.086	0.018	0.123	0.028
**12**	0.186	0.102	0.500	0.359	0.046	0.016	0.082	0.013
**24**	0.038	0.014	0.168	0.148	0.012	0.005	0.033	0.007

Table S2. Mean plasma concentration (μg/ml) ENR and CPR HPLC analyses after *per os* ENR application and ENR+propolis (PP) application.

Time (h)	ENR		ENR+PP		CPR		CPR+PP	
	Mean	SD	Mean	SD	Mean	SD	Mean	SD
**0**	0	0	0	0	0	0	0	0
**0.083**	0.290	0.169	0.997	0.986	0	0	0	0
**0.25**	0.554	0.365	1.407	1.079	0.020	0.022	0.022	0.011
**0.5**	1.198	0.386	1.690	1.220	0.094	0.060	0.046	0.024
**1**	1.663	0.465	1.989	1.005	0.218	0.116	0.090	0.032
**2**	2.255	0.436	2.448	0.680	0.318	0.105	0.142	0.039
**4**	2.063	0.427	2.283	0.424	0.354	0.096	0.184	0.061
**8**	1.234	0.548	1.909	0.291	0.229	0.039	0.187	0.086
**12**	0.707	0.528	1.435	0.312	0.140	0.044	0.154	0.080
**24**	0.233	0.243	0.699	0.211	0.050	0.028	0.084	0.022

Table S3. Mean (±SD) pharmacokinetic parameters of enrofloxacin following *per os* administration of enrofloxacin (ENR, 20 mg/kg) and intramuscular administration of enrofloxacin (ENR, 10 mg/kg) administered in rabbits (n=6).

	Per os	**Intramuscular**
**Parameters**	**ENR**	**ENR**
$T_{1/2\lambda z}$ (h)	5.93±2.51	2.88±0.59*
T_{max} (h)	2.00±1.10	0.47±0.08*
C_{max} (μg/ml)	2.38±0.34	2.98±0.35*
C_{max} (μg/ml) (dose-normalized)	1.19±0.17	2.98±0.35**
T_{last} (h)	24.00±0.00	24.00±0.00
C_{last} (μg/ml)	0.23±0.24	0.04±0.01
$AUC_{0-\infty}$ (μg.h/ml)	26.11±12.44	12.59±2.34*
$AUC_{0-\infty}$ (μg.h/ml) (dose-normalized)	13.05±6.22	12.59±2.34
$AUMC_{0-\infty}$ (μg.h^2/ml)	272.94±237.10	59.21±18.78
$AUMC_{0-\infty}$ (μg.h^2/ml) (dose-normalized)	136.47±118.55	59.21±18.78
$MRT_{0-\infty}$ (h)	8.96±3.82	4.63±0.69*

$T_{1/2\lambda z}$: Terminal half-life, T_{max}: Time to reach peak plasma concentration, C_{max}: Peak plasma concentration, T_{last}: Time to last detectable concentration, C_{last}: Last detectable concentration, $AUC_{0-\infty}$: Area under the concentration-time curve from zero up to ∞ with extrapolation of the terminal phase, $AUMC_{0-\infty}$: Area under the first moment of the concentration-time curve from zero up to ∞ with extrapolation of the terminal phase, $MRT_{0-\infty}$: Mean residence time from zero up to ∞ with extrapolation of the terminal phase. **$P<0.01$; *$P<0.05$ (statistically different from per os enrofloxacin)

Table S4. Mean (±SD) pharmacokinetic parameters of enrofloxacin following *per os* administration of enrofloxacin (ENR, 20 mg/kg) with propolis (PP, 100 mg resin/kg) and intramuscular administration of enrofloxacin (ENR, 10 mg/kg) with propolis (PP, 100 mg resin/kg) administered in rabbits (n=6).

	Per os	Intramuscular
**Parameters**	**ENR+PP**	**ENR+PP**
$T_{1/2\lambda z}$ (h)	11.75±3.20	5.64±1.63**
T_{max} (h)	3.08±2.65	0.52±0.29
C_{max} (μg/ml)	2.65±0.65	2.90±0.45
C_{max} (μg/ml) (dose-normalized)	1.33±0.33	2.90±0.45**
T_{last} (h)	24.00±0.00	24.00±0.00
C_{last} (μg/ml)	0.70±0.21	0.17±0.15**
$AUC_{0-\infty}$ (μg.h/ml)	48.91±11.53	21.18±8.86**
$AUC_{0-\infty}$ (μg.h/ml) (dose-normalized)	24.46±5.77	21.18±8.86
$AUMC_{0-\infty}$ (μg.h²/ml)	876.29±399.59	180.15±143.37**
$AUMC_{0-\infty}$ (μg.h²/ml) (dose-normalized)	438.14±199.80	180.15±143.37*
$MRT_{0-\infty}$ (h)	17.26±4.55	7.72±2.53**

$T_{1/2\lambda z}$: Terminal half-life, T_{max}: Time to reach peak plasma concentration, C_{max}: Peak plasma concentration, T_{last}: Time to last detectable concentration, C_{last}: Last detectable concentration, $AUC_{0-\infty}$: Area under the concentration-time curve from zero up to ∞ with extrapolation of the terminal phase, $AUMC_{0-\infty}$: Area under the first moment of the concentration-time curve from zero up to ∞ with extrapolation of the terminal phase, $MRT_{0-\infty}$: Mean residence time from zero up to ∞ with extrapolation of the terminal phase. **P<0.01; *P<0.05 (statistically different from per os ciprofloxacin)

Table S5. Mean (±SD) pharmacokinetic parameters of ciprofloxacin following *per os* administration of enrofloxacin (ENR, 20 mg/kg) and intramuscular administration of enrofloxacin (ENR, 10 mg/kg) administered in rabbits (n=6).

	Per os	Intramuscular
**Parameters**	**CPR**	**CPR**
$T_{1/2\lambda z}$ (h)	7.22±2.34	4.97±1.34
T_{max} (h)	4.76±1.63	2.33±0.82*
C_{max} (μg/ml)	0.36±0.09	0.21±0.03*
C_{max} (μg/ml) (dose-normalized)	0.18±0.05	0.21±0.03
T_{last} (h)	24.00±0.00	24.00±0.00
C_{last} (μg/ml)	0.05±0.03	0.01±0.01*
$AUC_{0-\infty}$ (μg.h/ml)	4.68±0.98	1.88±0.31**
$AUC_{0-\infty}$ (μg.h/ml) (dose-normalized)	2.34±0.49	1.88±0.31
$AUMC_{0-\infty}$ (μg.h²/ml)	55.45±27.43	14.87±5.22*
$AUMC_{0-\infty}$ (μg.h²/ml) (dose-normalized)	27.72±13.72	14.87±5.22
$MRT_{0-\infty}$ (h)	11.39±3.82	7.75±1.45

$T_{1/2\lambda z}$: Terminal half-life, T_{max}: Time to reach peak plasma concentration, C_{max}: Peak plasma concentration, T_{last}: Time to last detectable concentration, C_{last}: Last detectable concentration, $AUC_{0-\infty}$: Area under the concentration-time curve from zero up to ∞ with extrapolation of the terminal phase, $AUMC_{0-\infty}$: Area under the first moment of the concentration-time curve from zero up to ∞ with extrapolation of the terminal phase, $MRT_{0-\infty}$: Mean residence time from zero up to ∞ with extrapolation of the terminal phase. **P<0.01; *P<0.05 (statistically different from per os ciprofloxacin).

Table S6. Mean (±SD) pharmacokinetic parameters of ciprofloxacin following *per os* administration of enrofloxacin (ENR, 20 mg/kg) with propolis (PP, 100 mg resin/kg) and intramuscular administration of enrofloxacin (ENR, 10 mg/kg) with propolis (PP, 100 mg resin/kg) administered in rabbits (n=6).

	Per os	**Intramuscular**
**Parameters**	**CPR+PP**	**CPR+PP**
$T_{1/2\lambda z}$ (h)	16.35±4.72	8.74±1.93**
T_{max} (h)	5.33±2.07	2.40±0.89*
C_{max} (μg/ml)	0.20±0.08	0.20±0.06
C_{max} (μg/ml) (dose-normalized)	0.10±0.04	0.20±0.06*
T_{last} (h)	24.00±0.00	24.00±0.00
C_{last} (μg/ml)	0.08±0.02	0.03±0.01**
$AUC_{0-\infty}$ (μg.h/ml)	5.23±1.41	2.71±0.45**
$AUC_{0-\infty}$ (μg.h/ml) (dose-normalized)	2.62±0.70	2.71±0.45
$AUMC_{0-\infty}$ (μg.h^2/ml)	127.97± 34.55	34.97±6.60**
$AUMC_{0-\infty}$ (μg.h^2/ml) (dose-normalized)	63.99±17.18	34.97±6.60**
$MRT_{0-\infty}$ (h)	24.99±6.51	13.08±2.74**

$T_{1/2\lambda z}$: Terminal half-life, T_{max}: Time to reach peak plasma concentration, C_{max}: Peak plasma concentration, T_{last}: Time to last detectable concentration, C_{last}: Last detectable concentration, $AUC_{0-\infty}$: Area under the concentration-time curve from zero up to ∞ with extrapolation of the terminal phase, $AUMC_{0-\infty}$: Area under the first moment of the concentration-time curve from zero up to ∞ with extrapolation of the terminal phase, $MRT_{0-\infty}$: Mean residence time from zero up to ∞ with extrapolation of the terminal phase. **P<0.01; *P<0.05 (statistically different from per os ciprofloxacin with propolis)
